# Earthquakes and Taliban Decrees: The Plight of Afghan Women and Children

**DOI:** 10.7759/cureus.48663

**Published:** 2023-11-11

**Authors:** Mirwais Ramozi, Yudai Kaneda, Hosain Barati, Akihiko Ozaki, Sayed H Mousavi

**Affiliations:** 1 Faculty of Medicine, Kateb University, Kabul, AFG; 2 School of Medicine, Hokkaido University, Sapporo, JPN; 3 Department of Breast and Thyroid Surgery, Jyoban Hospital of Tokiwa Foundation, Iwaki, JPN

**Keywords:** maternal and children health, taliban, global healthcare systems, earthquakes, afghanistan

## Abstract

Following the Taliban's takeover in August 2021, Afghanistan confronts compounded challenges from both seismic political shifts and catastrophic natural events. Earthquakes in Khost and Herat provinces have resulted in thousands of casualties, with the majority being women and children, highlighting structural vulnerabilities of Afghan homes made from sun-dried bricks. Concurrently, the Taliban's restriction on women's participation in non-governmental organizations (NGOs) has strained essential health and aid services. This situation is exacerbated by international decisions to reduce aid allocations. This convergence of crises has disproportionately affected women and children, with potential surges in malnutrition, diseases, and child marriages. This scenario underscores the urgent need for the global community to prioritize humanitarian considerations over political disagreements, ensuring aid reaches the vulnerable and NGOs can operate amidst ongoing challenges.

## Editorial

Since the Taliban's swift takeover in August 2021, Afghanistan has been beset not only by profound political shifts but also by devastating natural calamities, exacerbating an already dire situation. In June of the previous year, a magnitude 5.9 earthquake struck the eastern province of Khost, tragically killing over 1,000 people [1]. More recently, the western province of Herat has experienced a series of magnitude 6 earthquakes since October 7, 2023 [2]. The Taliban's interim administration acknowledges 2,445 fatalities, but as many remain unaccounted for, the true death toll may yet rise [2]. United Nations International Children's Emergency Fund (UNICEF) stressed that over 90% of these victims were also women and children, because most men were away at work when the initial quake struck, leaving women and children at home [2]. Indeed, it has been noted that a significant proportion of Afghan dwellings are constructed from sun-dried bricks, similar to those implicated in the extensive damage during the Nepal earthquake in 2015, which likely contributed to the magnitude of the devastation experienced [1].

These natural disasters compound the challenges arising from the Taliban's decree prohibiting women from working in non-governmental organizations (NGOs) [3]. So far, given the Afghan government's limited capacity and resources, NGOs have been instrumental in tackling the country's healthcare challenges. Women, notably, have been central to this effort, participating in services like maternal and child healthcare, community-based activities, nutrition, and psychological support. Therefore, one of the pressing issues is the high infant and maternal mortality rates compounded by the Taliban's recent decree prohibiting women from working in NGOs [4].

Moreover, with the Taliban's decree excluding women from these NGOs, international funders and countries have limited their cooperation, further straining an already delicate situation. The situation was further aggravated when the UK government allocated only £1m out of the promised £7m to Save the Children, and the German NGO Welthungerhilfe suspended its work. Notably, as a result of the decree, 83% of NGOs had to suspend some or all of their operations by January 12 [5].

The combined effects of political decisions and natural disasters are particularly harsh on already marginalized women and children. Not only is there a potential link to the already severe issue of child malnutrition and an increase in marriages, but it may also impede the progress of women in society, exacerbate mental health problems, and deepen the disconnect from international societal trends [5].

In conclusion, the intertwining of political edicts from the Taliban and devastating natural disasters have created a critical juncture for Afghanistan. Sanctions and aid cuts, though meant to penalize the Taliban, inadvertently have a harsh impact on Afghan women and children. This situation serves as a poignant call to the international community, urging them not to let political or natural crises imperil the lives of the most vulnerable. It is imperative to separate humanitarian aid from political disputes, negotiate feasible solutions with the Taliban, and continue the essential work of NGOs, especially in light of the recurring natural disasters.
